# First Laboratory Detection of N^13^CO^–^ and Semiexperimental Equilibrium Structure of the NCO^–^ Anion

**DOI:** 10.1021/acs.jpca.2c00313

**Published:** 2022-03-14

**Authors:** Luca Dore, Luca Bizzocchi, Valerio Lattanzi, Mattia Melosso, Filippo Tamassia, Michael C. McCarthy

**Affiliations:** † Dipartimento di Chimica “Giacomo Ciamician”, Università di Bologna, Via Selmi 2, I-40126 Bologna, Italy; ‡ Center for Astrochemical Studies, Max Planck Institut für Extraterrestrische Physik Gießenbachstraße 1, D-85748 Garching bei München, Germany; § Scuola Superiore Meridionale, Università di Napoli Federico II, Largo San Marcellino 10, I-80138 Naples, Italy; ∥ Dipartimento di Chimica Industriale “Toso Montanari”, Università di Bologna, Viale Risorgimento 4, I-40136 Bologna, Italy; ⊥ Center for Astrophysics|Harvard & Smithsonian, 60 Garden St., Cambridge, Massachusetts 02138, United States

## Abstract

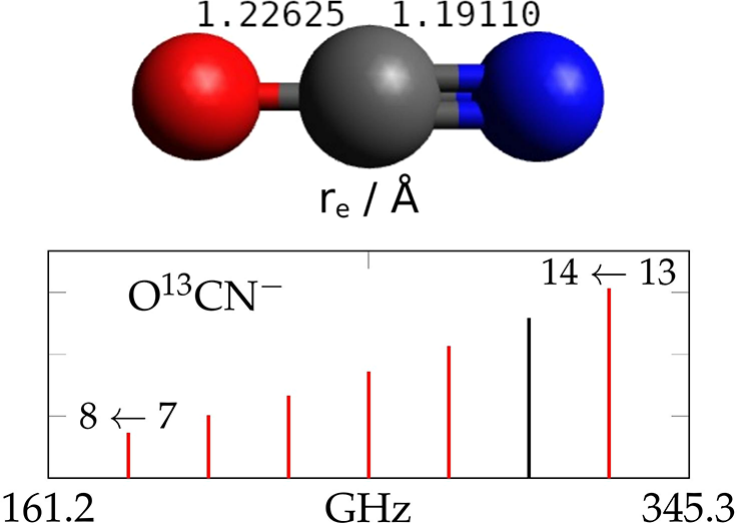

The cyanate anion (NCO^–^) is a species of considerable astrophysical relevance. It is widely believed to be embedded in interstellar ices present in young stellar objects but has not yet been detected in the dense gas of the interstellar medium. Here we report highly accurate laboratory measurements of the rotational spectrum of the N^13^CO^–^ isotopologue at submillimeter wavelengths along with the detection of three additional lines of the parent isotopologue up to 437.4 GHz. With this new data, the rotational spectrum of both isotopologues can be predicted to better 0.25 km s^–1^ in equivalent radial velocity up to 1 THz, more than adequate for an astronomical search in any source. Moreover, a semiexperimental equilibrium structure of the anion is derived by combining the experimental ground-state rotational constants of the two isotopologues with theoretical vibrational corrections, obtained by using the coupled-cluster method with inclusion of single and double excitations and perturbative inclusion of triple excitations (CCSD(T)). The estimated accuracy of the two bond distances is on the order of 5 × 10^–4^ Å: a comparison to the values obtained by geometry optimization with the CCSD(T) method and the use of a composite scheme, including additivity and basis-set extrapolation techniques, reveals that this theoretical procedure is very accurate.

## 1 Introduction

The cyanate ion is the conjugate base of cyanic (HOCN) and isocyanic (HNCO) acids and serves as a well-known pseudohalide anion in salts which are employed in organic synthesis. It has also a biological role since it is a human metabolite. NCO^–^ is almost certainly embedded in interstellar ices^1^ with experimental and theoretical studies strongly supporting the argument that the so-called XCN^2^ feature at 2165 cm^–1^ (4.62 μm) observed toward various young stellar objects is due to this ion.^3−6^ It has been proposed that NCO^–^ can be formed on icy grain mantles from the isocyanic acid, either by ionization of the acid interacting with NH_3_ solvated by H_2_O molecules^7^ or by thermal processing.^8^ Additional mechanisms, involving radicals (NH_2_/NH + CO) and suprathermal oxygen atoms (O + CN^–^), have been also put forth,^5^ as has a suggestion that NCO^–^ is efficiently formed by the interaction of cosmic rays with CO–NH_3_ ices.^9^

Experiments of proton irradiation and UV photolysis of nitriles frozen on H_2_O ice, performed to explore the chemistry induced by far-UV photons and ionizing radiation, led to the formation NCO^–^, whereas under anhydrous conditions isonitriles were formed.^10^ On this basis, Hudson and Moore^10^ argue that the cyanate ion is a possible stable radiation product in Titan or cometary ices where both nitriles and H_2_O are present. In addition, its production by electron bombardment of an ice mixture of H_2_O, CO_2_, CH_4_, NH_3_, and CH_3_OH led Bergantini et al.^11^ to postulate a relevance of cyanate in the chemistry of Enceladus.

Although NCO^–^ does not react by associative detachment with H_2_,^12^ it has yet to be detected in molecular clouds. Presumably the absence of NCO^–^ there points to the lack of an efficient gas-phase formation route. Yurtsever et al.,^13^ for example, discarded the possibility of radiative electron attachment to the NCO radical because of the high electron affinity of the anion (3.609 eV^14^) and its small size, which makes dissipation of the large amount of energy needed for the stabilization inefficient.

The rotational spectrum of NCO^–^ was first reported by Lattanzi et al.,^15^ who measured its spectrum both in a supersonic molecular beam and in a low-pressure glow discharge between 23 and 368 GHz. In addition to the fundamental (*J*′ = 1 → 0) transition at 23 GHz, which displayed the characteristic triplet of the nitrogen hyperfine structure, nine harmonically related lines with *J* = 6–15 were also detected. The carrier of these lines can be assigned with certainty to NCO^–^ since the derived rotational constant, albeit much more precise, is in very good agreement with early infrared measurements of Gruebele et al.^16^ In that study, a total of 132 IR transitions in the ν_3_ (CN stretching) fundamental and corresponding bending and stretching hot bands were reported, and the equilibrium rotational constant derived on the basis of the all three normal coordinates. However, assignments of two hot bands were subsequently questioned, first by Botschwina et al.^17^ and later by Pak et al.,^18^ who proposed a reassignment. As a result, the only reliable vibration–rotation interaction constant from the IR analysis is α_3_, which arises from the CN stretch.

The present work was undertaken with the intention of determining a precise molecular structure of NCO^–^. Our efforts have focused on the millimeter-wave spectrum of the C-13 isotopologue, in addition to extending measurements of the parent isotopologue into the submillimeter-wave region. With the rotational constants available for two isotopologues, a semiexperimental equilibrium structure of the anion was obtained by using vibrational corrections from quantum-chemical calculations.

## 2 Methods

### 2.1 Experimental Section

#### 2.1.1 Parent Isotopologue

The submillimeter-wave rotational lines of the cyanate ion reported in this work were observed in Bologna with a frequency-modulated spectrometer^19^ equipped with a discharge cell made of a Pyrex tube, 3.25 m long and of 5 cm diameter, with two cylindrical hollow electrodes 25 cm in length at either end. The radiation source was a frequency quadrupler, which consists of two doublers in cascade (RPG—Radiometer Physics GmbH) driven by a Gunn diode oscillator (J. E. Carlstrom Co.) operating between 75 and 115 GHz. Two phase-lock loops allow the stabilization of the Gunn oscillator with respect to a frequency synthesizer, which is locked to a 5 MHz rubidium standard. Frequency modulation of the radiation is obtained by sine-wave modulating at 6 kHz, the reference signal of the wide-band Gunn synchronizer (total harmonic distortion less than 0.01%). The signal, detected by a liquid-helium-cooled InSb hot electron bolometer (QMC Instr. Ltd. type QFI/2), is demodulated at 2*f* by a lock-in amplifier.

**Figure 1:**
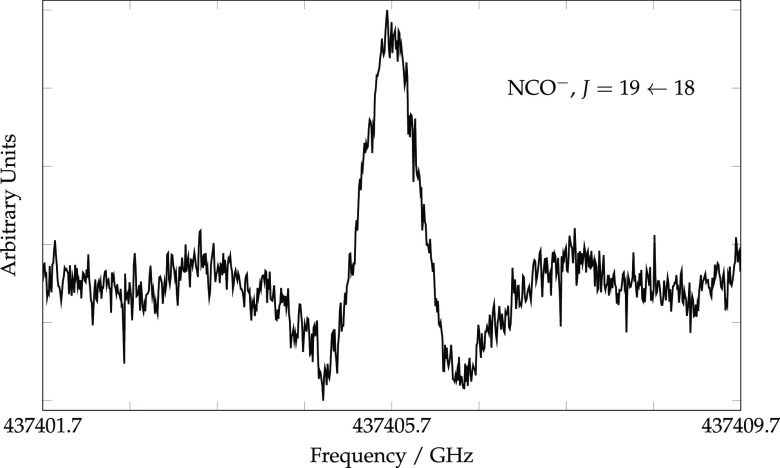
*J* = 19 ← 18 rotational transition of NCO^–^ with the background subtracted; the total integration time was 565 s for a scan at 3.24 MHz/s with a time constant of 3 ms.

The best conditions for NCO^–^ were found by maximizing the production of HNCO via its *J*_*K*_*a*_,*K*_*c*__ = 53_0,53_ ← 52_1,52_ transition near 368 GHz.^a^ Using a DC-discharge operating at room temperature, we combined a 5:2 mixture (7 mTorr, 0.9 Pa) of humid air and CO with He buffer gas for a total pressure of about 15 mTorr (2.0 Pa), and the discharge current varied between 20 and 40 mA. Anion production was slightly improved by the application of an axial magnetic field (∼55 G) along the entire length of the discharge cell (which increases the length of the negative glow region), unlike protonated CO or N_2_, where cation production in the negative glow is greatly enhanced with respect to that achieved in the positive column.^20^ Not surprisingly, the formation mechanisms of cations and anions are markedly different: for cations, ionizing collisions of the magnetically confined electrons are important, while NCO^–^ is more likely produced by dissociative electron attachment to HNCO.^15^ Our measurements, although indirect, support this hypothesis, in that there is a close correlation between the production of HNCO and NCO^–^, regardless of the precursor combination used. A sample spectrum of the *J* = 19 ← 18 transition is shown in Figure 1. Because a signal-to-noise ratio of less than 10 was achieved in 10 m of integration and the integrated line strength of the transition is fairly high (5 × 10^–2^ nm^2^ MHz), the steady-state abundance of the anion under our most favorable discharge conditions is low.

Finally, the accuracy of frequency measurements of NCO^–^ was carefully checked since line centers can be shifted by the Doppler effect due to the drift velocity of molecular ions produced in the positive column of a DC glow discharge.^21^ Frequency measurements of the test transition *J* = 16 ← 15 at 368.372 GHz, previously observed by Lattanzi et al.,^15^ did not yield a statistically significant difference in the line frequency when the polarity of the cell electrodes was reversed. This test does not preclude the possibility of a small Doppler shift; rather, it may simply be obscured by the poor signal-to-noise ratio.

#### 2.1.2 C-13 Isotopologue

The same free space absorption spectrometer in Cambridge^15^ that was used to observe the millimeter-wave spectrum of NCO^–^ was also employed to detect the C-13-containing isotopologue, N^13^CO^–^. Radiation was produced by frequency multiplication from one of several phase-locked Gunn oscillator that operate in the region 70–145 GHz and fed into a double-pass absorption cell 3 m in length. The signal, modulated at *f* = 95 kHz, was detected by a liquid-helium-cooled InSb hot-electron bolometer and then demodulated at 2*f* with a lock-in amplifier.

Unlike the original study, ^13^CO was used instead of CO and was combined with N_2_ and Ar in a 1:2:2 mixture at a total pressure of 10 mTorr (1.3 Pa); the cell was kept at about 290 K, and the discharge current was set at 30 mA. Although no H-containing precursors were used, enough trace hydrogen was present to readily detect HN^13^CO, which in turn could dissociate to N^13^CO^–^ after electron attachment in the low-density plasma. Evidence implicating this production scheme came from detection of anion signal even after the precursor flow was switched off but while the discharge was maintained with Ar buffer gas alone. The presence of N^13^CO^–^ under these conditions likely comes about simply because isocyanic acid is metastable and persists in the cell for some time after its formation.

**Figure 2:**
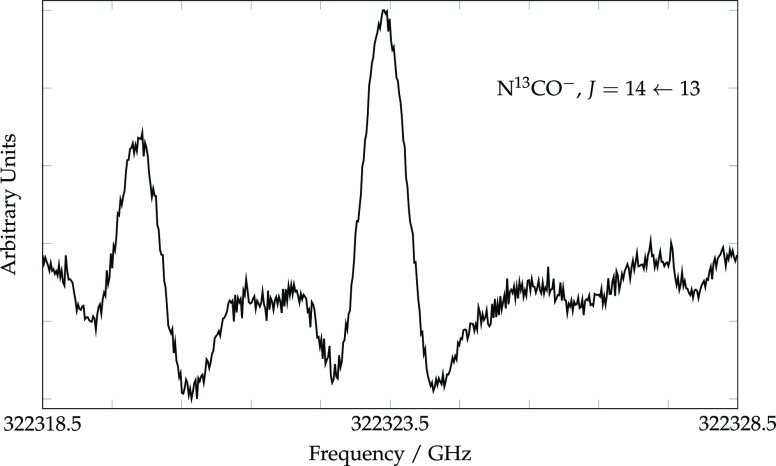
*J* = 14 ← 13 rotational transition of N^13^CO^–^ with the background subtracted; the total integration time was 341 s for the average of six sweeps in the forward and reverse directions. The feature at lower frequency is from an unassigned molecule present in the glow discharge.

Because of the small change on the moment of inertia upon substitution of the C atom, a reliable prediction of the rotational constant of the C-13 isotopologue was calculated by scaling the theoretical *B* constant of N^13^CO^–^ by the ratio of the theoretical and experimental rotational constants for the parent species. The first transition sought was the *J* = 14 ← 13 at 322.323 GHz, and indeed a discharge-dependent line was soon observed (see Figure 2). Nevertheless, the detection of additional harmonically related lines at higher and lower frequency, which was needed to confirm the identification, proved challenging because even modestly strong lines from unrelated species easily obscure the relative weak lines of N^13^CO^–^. For example, the *J* = 15 ← 14 of N^13^CO^–^ lies very close in frequency to a strong transition of H^13^CN and could not be measured.

### 2.2 Computational Details

The CFOUR program package^22^ has been employed to obtain an ab initio estimate of the equilibrium (*r*_e_) structure and the vibrational corrections needed to derive the semiexperimental equilibrium (*r*_SE_) structure. The coupled-cluster method with inclusion of single and double excitations and perturbative inclusion of triple excitations, CCSD(T),^23^ has been used. For the electrons of the three atoms, correlation-consistent basis sets of Dunning (cc-pV*n*Z)^24^ have been applied, augmented by diffuse functions (aug-cc-pV*n*Z)^25^ for a proper description of the anion.

An automatized composite scheme implemented in CFOUR has been adopted for the geometry optimization.^26^ Briefly, the method first uses basis-set extrapolation at the Hartree–Fock (HF) level in which extrapolation of the energy gradient to the complete basis set limit is achieved by using three basis sets of the aug-cc-pV*n*Z hierarchy with *n* = T, Q, 5. Then, extrapolation of the correlation-energy contribution was performed with the two basis sets aug-cc-pVTZ and aug-cc-pVQZ. In the framework of the additivity scheme, the correlation energy gradient is added to the HF term. With the previous two terms obtained in the frozen-core (fc) approximation, a third energy gradient accounting for inner-shell correlation contributions has to be added, and for this purpose we performed all-electron and the corresponding fc calculations at the CCSD(T) level with the basis set aug-cc-pCVTZ.

Vibrational corrections to the equilibrium rotational constants have been computed within a second-order vibrational perturbation theory (VPT2) treatment using the full cubic force field together with the semidiagonal part of the quartic force field evaluated at the CCSD(T)/aug-cc-pV*n*Z level where three calculations with *n* = T, Q, 5 have been performed to check for accuracy. The presence of a Fermi resonance between the *v*_1_ = 1 and *v*_2_ = 2 states has been considered by removing the resonance denominator in the sum of the first-order vibration–rotation interaction constants.

## 3 Results and Discussion

### 3.1 Rotational Spectra

Using the spectroscopic constants of Lattanzi et al.,^15^ we observed three higher-frequency rotational lines of the main isotopologue at 391.4, 414.14, and 437.4 GHz, with *J* = 16, 17, and 18 (see Table 1). Attempts to detect still higher frequency lines (e.g., the *J* = 20 ← 19 transition) was unsuccessful, in large part because of a rapid drop-off of the millimeter-wave power at shorter wavelengths. At high rotation quantum numbers, hyperfine structure due to the ^14^N nucleus was blended in a single peak, and the unperturbed line frequency was recovered by a line shape analysis of the spectral profile modeled as a single Voigt profile.

**Table 1:** Measured Rotational Transition Frequencies of NCO^–^

transition			
*J*′ ← *J*	*F*′ ← *F*	frequency^a^ (MHz)	residual (kHz)
1 ← 0	1 ← 1	23027.659^b^	–1
1 ← 0	2 ← 1	23027.969^b^	0
1 ← 0	0 ← 1	23028.432^b^	0
7 ← 6	c	161189.304^b^	10
8 ← 7	c	184214.146^b^	–1
9 ← 8	c	207238.157^b^	31
10 ← 9	c	230261.101^b^	–18
12 ← 11	c	276303.702^b^	–12
13 ← 12	c	299323.109^b^	12
14 ← 13	c	322341.060^b^	3
15 ← 14	c	345357.465^b^	–21
16 ← 15	c	368372.276^b^	3
17 ← 16	c	391385.317	8
18 ← 17	c	414396.497	11
19 ← 18	c	437405.683	–10

a The estimated 1σ uncertainties are 2 kHz for the *J* = 1 ← 0 transition and 20 kHz for the millimeter- and submillimeter-wave lines.

b From ref (15).

c Hyperfine splitting unresolved.

Using the standard frequency expression of the rotational transition *J* + 1 ← *J*
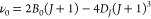
1and the experimental transition frequencies, we determined B_0_ and *D*_*J*_, the ground-state rotational and quartic centrifugal distortion constants, respectively, using a weighted least-squares procedure.^27^ Because hyperfine structure was resolved in the *J* = 1 ← 0 measurements in ref (15), the quadrupole coupling (*eqQ*) constant of the N nucleus (spin quantum number *I* = 1) can also be derived according to the expression
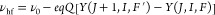
2where *F* is the quantum number associated with the total angular momentum (*F⃗* = *J⃗* + *I⃗*), and the Casimir function *Y*(*J*,*I*,*F*) is given by
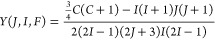
3with *C*(*J,I,F*) = *F*(*F* + 1) – *I*(*I* + 1) – *J*(*J* + 1).

The Pickett’s SPFIT fitting program^27^ was used to simultaneously model the nitrogen hyperfine structure at low frequency and centrifugal analysis at high frequency in a combined fit, where each feature is weighted by the inverse square of the uncertainty of its transition frequency. The fit residuals and the best-fit spectroscopic constants of NCO^–^ derived in this fashion are reported in Tables 1 and 2, respectively. For completeness, these constants are compared to those reported by Lattanzi et al.^15^ Of particular note is the increased precision of *D*_*J*_ which has been improved by roughly an order of magnitude here. In turn, still higher *J* lines can now be predicted to better than 0.08 km s^–1^ in equivalent radial velocity up to 1 THz, more than adequate for searches in the most quiescent molecular clouds.

**Table 2:** Spectroscopic Constants of NCO^–^

constants	Lattanzi et al.	this work^a^	correlation matrix		
*B*_0_ (MHz)	11513.9683(8)	11513.96777(43)	1.000		
*D*_*J*_ (kHz)	4.561(2)	4.55907(87)	–0.885	1.000	
*eqQ* (MHz)	–1.0307(37)	–1.0302(37)	0.221	–0.196	1.000
rms_res_^b^ (kHz)		12.7			
σ^c^		0.724			

a Errors, as listed in the output of the SPFIT program,^27^ are reported in parentheses in units of the last quoted digits; standard errors of the fit parameters are obtained multiplying by σ.

b Rms error of residuals: .

c Fit standard deviation: .

For the C-13 isotopologue, in addition to the *J* = 14 ← 13 transition at 322.323 GHz, a total of five lines free from interloping or strong interfering features were ultimately detected in the range 160–300 GHz and unambiguously assigned to transitions with *J* = 7–11; these data are summarized in Table 3. Best-fit spectroscopic constants were determined in an analogous procedure as that used for the parent species sans the nitrogen hyperfine structure since the fundamental rotational transition of N^13^CO^–^ was not measured in ref (15). Table 3 provides the residuals of the fit while the best-fit spectroscopic constants are given in Table 4 for N^13^CO^–^.

**Table 3:** Measured Rotational Transition Frequencies of N^13^CO^–^

transition *J*′ ← *J*	frequency^a^ (MHz)	residual (kHz)
8 ← 7	184204.055	0
9 ← 8	207226.785	15
10 ← 9	230248.488	–11
11 ← 10	253269.142	8
12 ← 11	276288.544	–20
14 ← 13	322323.382	8

a The estimated 1σ uncertainty is 20 kHz.

**Table 4:** Spectroscopic Constants of N^13^CO^– ^a

constant	value	correlation matrix	
*B*_0_ (MHz)	11513.33735(117)	1.000	
*D*_*J*_ (kHz)	4.5620(42)	–0.947	1.000
rms_res_ (kHz)	12.3		
σ	0.613		

a Notes of Table 2 apply to this table as well.

### 3.2 Vibrational Corrections

Table 5 reports the contributions of zero-point vibration motion to the rotational constant for both NCO^–^ and N^13^CO^–^ by using three correlation-consistent Dunning’s basis sets. The differences in the derived values for the three basis sets is quite small, amounting to at most 1.8%. A particularly good test of the accuracy of the calculations is the theoretical values of α_3_, the largest vibration–rotation interaction constant, relative to the experimental value derived from the IR spectra.^16^ This discrepancy, at worst −4.4‰, quickly decreases as the size of the basis set increases: for the aug-cc-pV5Z basis set it is −0.04%, suggesting vibrational contributions can likely be calculated to an accuracy well within 0.15%.

**Table 5:** Calculated Vibrational Corrections for NCO^–^ and N^13^CO^–^

	NCO^–^			N^13^CO^–^
basis set	*B*_e_ – *B*_0_ (MHz)	α_3_ (cm^–1^)	diff^a^ (‰)	*B*_e_ – *B*_0_ (MHz)
aug-cc-pVTZ	46.5658	0.00296761	–4.4	45.9527
aug-cc-pVQZ	46.6154	0.00297521	–1.8	45.9990
aug-cc-pV5Z	47.3810	0.00297935	–0.4	46.7460

a Difference with respect to the experimental value of the vibration–rotation interaction constant α_3_ (= 0.0029806(22) cm^–1^ from ref (16)).

Table 6 reports the semiexperimental equilibrium *B*_e_ rotational constant for the two isotopologues studied here: it is derived from the experimental ground state constant, *B*_0_, plus the *ab initio* calculated vibrational correction, *B*_e_ – *B*_0_. Although the latter term contributes only for 0.4% to *B*_e_, its precision with respect to that of *B*_0_ is large and therefore dominates the resulting uncertainty of *B*_e_.

**Table 6:** Semiexperimental Equilibrium Rotational Constants of NCO^–^ and N^13^CO^–^

	NCO^–^	N^13^CO^–^
*B*_e_ (MHz)	11561.3488	11560.0833
rel accuracy^a^	6 × 10^–6^	

a Conservative value, estimated assuming an accuracy of 0.15% for vibrational corrections.

### 3.3 Semiexperimental Equilibrium Structure

Assuming a linear geometry for NCO^–^, the relationship between the moment of inertia and the two bond distances, *r*_NC_ and *r*_CO_, is the following (see Table 13.1 of ref (28)):
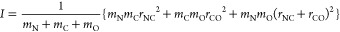
where *m*_α_ denotes the mass of the atom α.^b^ From the values of the moments of inertia of NCO^–^ and N^13^CO^–^ it is thus possible to determine the two bond lengths. However, this solution is poorly constrained when substitution is very close to the center of mass, and the value of the moment of inertia changes very little. Nevertheless, the stability of the solution can be trivially tested by artificially varying the rotational constant from the experimental value by a small amount. For example, increasing *B*_e_ of N^13^CO^–^ by 50 kHz (4 × 10^–4^% or a difference of about 50σ relative to our best-fit value) leads to an increase of *r*_NC_ and a decrease of *r*_CO_ of only ∼2 × 10^–3^ Å. In addition, the derived structure can be compared to those obtained purely from high-level quantum chemistry calculations. As noted in Table 7, the experimental bond lengths and those calculated theoretically are closely consistent. Based on these comparisons, the bond distances derived from the semiexperimental *B*_e_ constants appear to be determined to high accuracy.

**Table 7:** Molecular Structure of NCO^–^

		bond lengths (Å)	
	level of theory	N–C	C–O
semiexperimental		1.19110	1.22625
theory			
Botschwina et al., 1995	CCSD(T)/138cGTOs^a^	1.1917	1.2284
Pak et al., 1997	CCSD(T)/6s5p3d2f^b^	1.1934	1.2306
Prasad, 2004	*fv*-CASSCF/6s4p2d2f^c^	1.199	1.231
Léonard et al., 2010	CCSD(T)/aug-cc-pV5Z^d^	1.1928	1.2293
this work	CCSD(T)/aug-cc-pV*n*Z^e^	1.1898	1.2266

a Reference (17), with all electrons correlated.

b Reference (18), 150 cGTOs.

c Reference (30).

d Reference (31).

e For the composite scheme employed see text.

On the whole, inspection of Table 7 suggests the CCSD(T) method is the most accurate in calculating bond lengths of a triatomic linear molecule containing first and second row elements, presumably because of its ability to treat electron correlation effects. In addition, basis-set extrapolation, combined with additivity schemes, improves accuracy in geometry optimization. For NCO^–^, differences between the theoretical and semiexperimental bond lengths are about 1 mÅ or less: −1.3 × 10^–3^ and +0.3 × 10^–3^ Å for *r*_NC_ and *r*_CO_, respectively.

## 4 Conclusions

High-resolution spectroscopic observations of the cyanate anion are limited to the ground-state rotational spectrum and the ν_3_ fundamental and corresponding bending and stretching hot bands of NCO^–^. In addition to higher-frequency rotational measurements of the normal isotopic species, this study adds the rotational spectrum of the isotopologue N^13^CO^–^ to this body of work. From the two rotational constants, the two bond lengths of this linear triatomic can be derived. Going beyond a vibrationally averaged structure, quantum-chemical calculations have to be performed to obtain the vibrational contributions to the *B* constants, which, otherwise, would require extensive spectroscopic work. The molecular structure retrieved by such a combination of experimental and theoretical data, termed the semiexperimental structure, is the main result of this paper.

The accuracy of the two derived bond distances is estimated in the order of 5 × 10^–4^ Å, based on an assumed underestimation of the vibrational correction to the rotational constants of about 0.15%. In addition, geometry optimization with the CCSD(T) method and the use of a composite scheme, including additivity and basis-set extrapolation techniques, are very accurate for this simple, closed-shell linear molecule.

## Footnotes

a Listed in the Cologne Database for Molecular Spectroscopy (https://cdms.astro.uni-koeln.de) at 368.094 GHz.

b For an updated atomic mass evaluation see ref (29).
